# Utility of Temporal Bone Computed Tomography in Pediatric Emergency Medicine

**DOI:** 10.5811/westjem.2021.11.52704

**Published:** 2022-02-09

**Authors:** Sarah Benyo, Darrin V. Benn, Robert A. Saadi, Linda Gangai, Kathryn E. Kasmire, Huseyin Isildak, Neerav Goyal

**Affiliations:** * The Pennsylvania State University, College of Medicine, Hershey, Pennsylvania; † University of North Carolina, Department of Otolaryngology – Head and Neck Surgery, Chapel Hill, North Carolina; ‡ University of Arkansas for Medical Sciences, Department of Otolaryngology – Head and Neck Surgery, Little Rock, Arkansas; § Penn State Health Milton S. Hershey Medical Center, Department of Emergency Medicine, Hershey, Pennsylvania; ¶ The Pennsylvania State University, College of Medicine, Department of Otolaryngology – Head and Neck Surgery, Hershey, Pennsylvania

## Abstract

**Objective:**

Temporal bone computed tomography (CT) requires a relatively high radiation dose to produce high-resolution images required to define surgical anatomy. In the acute setting, the need for this detailed evaluation of temporal bone pathology may not be required for nonsurgical management and clinical decision-making. We performed a retrospective review of the clinical characteristics and subsequent management of children who underwent CT of the temporal bone with the goal of optimizing clinical decision-making and mitigating the risks of radiation exposure in children.

**Methods:**

We included pediatric patients (<18 years of age) with International Classification of Diseases (9th or 10th revision) diagnoses consistent with otitis externa, otitis media, mastoiditis, head trauma, temporal bone fracture, and otalgia who were treated in the emergency department and underwent temporal bone CT from January 1, 2012–December 31, 2016. We collected data regarding the patients’ presenting symptoms, physical exam findings, indications for imaging, radiographic findings, disposition, and operative intervention within 30 days of imaging. Features of the suspected mastoiditis group were compared between operative and non-operative patients.

**Results:**

Over the four-year study period there were 96 temporal bone CTs. Most studies (70%) were associated with a subsequent inpatient admission. Common indications for imaging included evaluation of acute mastoiditis (55%) or trauma (41%). Of the 53 patients with concern for mastoiditis, 27 (51%) required otologic surgery. Two patients in the trauma group required surgical intervention, both for facial nerve decompression. In patients with suspected mastoiditis, mental status changes (P = 0.02), auricular proptosis (P = 0.05), and fluctuance (P = 0.02) were significantly more prevalent in the operative group; however, no other findings were significantly associated with operative intervention.

**Conclusion:**

Temporal bone CT is beneficial in guiding diagnosis and management of acute mastoiditis. We found that a majority of patients with suspected mastoiditis who underwent temporal bone CT ultimately required surgery or hospital admission. However, the potential for reduction in the use of CT still exists in this population. Fractures of the temporal bone typically do not require urgent operative intervention in the absence of complete facial nerve paralysis; thus, the utility of temporal bone CT in trauma evaluation may be limited.

## INTRODUCTION

Up to seven million children in the United States undergo computed tomography (CT) annually, which has been raised as a public health concern due to radiation exposure and increased lifetime cancer risk.^1^,^2^ For this reason, multiple algorithms have been developed to reduce the radiation dose associated with CT in the pediatric population.^3^,^4^ The “as low as reasonably achievable” (ALARA) concept addresses methods for reducing the amount of radiation in a child while maintaining reliability of the diagnostic modality. The ALARA recommendations include developing weight-based protocols, considering alternative non-radiation modalities, and discouraging repeat CT imaging.^3^ Furthermore, young children may require sedation for imaging, adding additional risks, time, and cost.^5^ Ultimately, the best method of harm reduction is to avoid performing CT that will not inform or alter clinical decision-making.

Temporal bone CT is used in the pediatric population to identify acute middle- and inner-ear pathologies, often in the setting of infectious or trauma evaluation.^6^,^7^ Due to the complex bony anatomy, CT of the temporal bone requires a slice thickness of <1.0 millimeter and a high signal-to-noise ratio to minimize artifact for optimal visualization, thus requiring a higher radiation dose compared to routine head CT.^8^ Reducing the radiation dose of temporal bone CT below literature-derived protocols while maintaining accurate detection of findings of middle- and inner-ear structures is an area of active research.^9^

The high resolution of temporal bone CT aids in surgical planning by identifying infectious destruction of bone or the precise location of a temporal bone fracture that may otherwise be missed on lower-resolution imaging protocols. In the setting of temporal bone fracture, operative intervention with facial nerve decompression is indicated for patients with immediate facial nerve paresis and progressive decline in electroneuronography (ENoG) functioning to less than 10% of the normal side.^10^ Operative intervention is often indicated for complicated mastoiditis, with extracranial and intracranial sequelae encountered in 13–38% of mastoiditis cases.^11^,^12^ However, the current criteria for diagnosing complicated mastoiditis are diverse, and there is a lack of consensus regarding the strategies for diagnosis and the role of CT in the pediatric population.^13^

The primary objective of our study was to characterize the use of temporal bone CT in the acute emergency setting and investigate the clinical utility of this imaging modality in diagnosis and management of acute infectious and traumatic pathology of the temporal bone in the pediatric population. Our goal was to identify patient characteristics and common indications for temporal bone CT at our institution and to determine the subsequent clinical and/or operative management for these patients. Recognizing the appropriate scenarios to order temporal bone CT may prevent unnecessary radiation exposure in children presenting with such pathologies.

**Population Health Research Capsule**

What do we already know about this issue?

*High-resolution computed tomography (CT) may be used in pediatric infectious and traumatic temporal bone etiologies, but radiation dose is a public health concern.*

What was the research question?

*What is the clinical utility of temporal bone CT in pediatric acute infectious and traumatic pathologies?*

What was the major finding of the study?

*Temporal bone CT is beneficial for acute mastoiditis, but its utility in trauma evaluation may be limited.*

How does this improve population health?

*We identify areas for potential reduction in the use of temporal bone CT, which may limit unnecessary radiation exposure in the pediatric population.*

## MATERIALS AND METHODS

Following institutional review board approval at Penn State Hershey Medical Center, we conducted a retrospective review of pediatric emergency department (ED) visits at our institution between January 1, 2012 – December 31, 2016, for all pediatric patients who underwent CT temporal bone imaging over the specified time period. Patients with a primary *International Classification of Disease*s, 9^th^ or 10^th^ revision, diagnosis consistent with otitis externa, otitis media, mastoiditis, head trauma, temporal bone fracture, or otalgia were included to limit our study to the use of CT temporal bone imaging in the acute evaluation of infectious and traumatic etiologies. We collected data regarding patients’ presenting signs/symptoms and admission type (inpatient vs emergency), indications for CT temporal bone imaging, radiologic findings, and operative procedures performed within 30 days of CT imaging. For patients with suspected mastoiditis, we compared the operative and non-operative groups. The chart abstractors were not blinded to the study hypothesis. Statistical significance was determined by Fisher’s exact test with α = 0.05 implemented via the “stats” package in R v. 3.2.2 (The R Foundation for Statistical Computing, Vienna, Austria).

## RESULTS

Within our patient cohort there were 96 temporal bone CTs. Most studies (N = 67; 70%) were associated with an inpatient admission, while the remaining patients were discharged from the ED (N = 29; 30%). The most common indications for imaging were evaluation of acute mastoiditis (N = 53; 55%) or trauma (N = 39; 41%). The otolaryngology service was consulted for 68 patients, representing 79% of our patient cohort.

Otalgia, otorrhea, and post-auricular swelling were the most common presenting symptoms among patients with concern for mastoiditis. Mastoid tenderness, auricular proptosis, tympanic membrane opacification, and otorrhea were the most commons signs. Of the 53 patients with concern for mastoiditis, five had CT head performed prior to CT temporal bone, and otolaryngology was consulted for 66% of all infectious patients (N = 35) (Table 1). There was a total of 27 otologic procedures among the cohort, which included myringotomy and tympanostomy tube insertion and mastoidectomy, with or without abscess incision and drainage.

To determine the utility of temporal bone CT for patients with infectious concerns, we compared patient-reported symptoms, physical examination signs, and radiographic findings between patients who required operative intervention and those who did not (Table 2). Presentation of altered mental status was significantly more prevalent in the operative group (N = 5) compared to the non-operative group (N = 0; *P* = 0.02). Also, proptosis (*P* = 0.05) and post-auricular fluctuance (*P* = 0.02) were more frequent among the operative group. Radiographic findings of complicated mastoiditis (ie, post-auricular abscess, Bezold’s abscess, sigmoid sinus thrombosis, intracranial abscess) were reported among 10 patients in the operative group, and no patients in the non-operative group (*P* <0.01). No other clinical or radiologic findings were statistically associated with operative intervention; however, there were two patients in the operative group with facial nerve paralysis, which is a clear indication for operative management in this setting of infection. As expected, otolaryngology was consulted for all patients in the operative group (N = 27) compared to 31% (N = 8) of the non-operative group.

Of the trauma patients (N = 39), the most common presenting otologic signs and symptoms included hemotympanum, bloody otorrhea, hearing loss, otalgia, mastoid tenderness, and otorrhea (Table 1). Seventy-four percent of trauma patients who had CT temporal bone also had CT head (N =29), and 85% had otolaryngology consults (N = 33). The most common final diagnoses among trauma patients based on radiographic results were temporal bone fracture and mastoid effusion without radiographic evidence of fracture (Table 1). Among those who had temporal bone CT in the setting of trauma, two patients had operative intervention, both for facial nerve decompression.

## DISCUSSION

Temporal bone CT provides detailed anatomic information regarding the middle ear and temporal bone at the expense of a relatively high radiation dose, which is particularly undesirable in the pediatric population. To improve quality of care, clinicians should carefully weigh the risk of radiation exposure to the potential benefit of CT temporal bone imaging. Our study demonstrates that temporal bone CT is useful for surgical planning in the setting of complicated mastoiditis; however, mastoiditis remains primarily a clinical diagnosis that often does not require high-resolution imaging.^14^–^16^ Traumatic fractures can often be presumed in the setting of air cell opacification on head CT without the need to visualize the fracture with a high-resolution image.^17^ Moreover, surgical intervention is typically not required in the absence of complete facial nerve paralysis.^15^,^18^ Therefore, temporal bone CT should not be a routine study in the workup of these patients, especially in the pediatric population.

**Figure 1:**
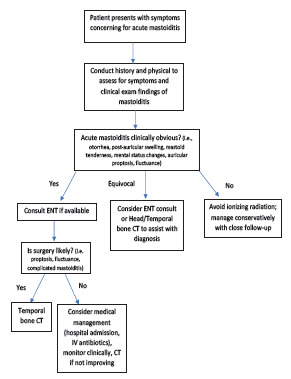
Proposed approach for guiding decision-making in pediatric patients with concern for acute infection of the temporal bone. *ENT*, ear nose throat; *CT*, computed tomography; *IV*, intravenous.

**Figure 2:**
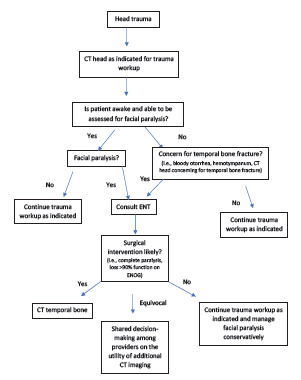
Proposed algorithm for guiding decision-making in pediatric trauma patients with concern for temporal bone fracture. *CT*, computed tomography; *ENT*, ear, nose, and throat; *ENoG*, electroneuronography.

Based on our findings, we have proposed approaches to obtaining temporal bone CT imaging between the specialties of emergency medicine and otolaryngology to maximize usefulness among pediatric patients with acute infectious concerns (Figure 1), as well as those with temporal bone fractures (Figure 2). In the setting of temporal bone fractures due to head trauma, it is possible that the patient will be sedated, intubated, or unable to follow commands. In such cases, assessment of facial paralysis may not be feasible and should be deferred until the patient is awake, and trauma evaluation should then proceed as indicated. However, it is important to note that if a patient is subsequently found to have a complete paralysis, the injury is treated as an immediate complete paralysis as opposed to delayed paralysis, and surgical intervention should be pursued.^19^,^20^

In our pediatric ED population, nearly half of patients who underwent CT to evaluate for mastoiditis required operative intervention. This indicates that half of patients who received temporal bone CT did not require surgery, potentially exposing these children to unnecessary ionizing radiation. While it is possible that patients may require a temporal bone CT for admission for medical management of suspected mastoiditis, our data does highlight the importance of identifying clinical findings that raise suspicion for surgical intervention (ie, mental status changes, proptosis, fluctuance) when deciding whether temporal bone CT is necessary.

The literature suggests that CT may be beneficial in confirming the diagnosis of mastoiditis in patients who do not present with a clear clinical picture.^7^ Future studies should examine scenarios in which CT can be avoided altogether in patients with acute infection and when head CT alone or other lower-radiation temporal bone CT techniques may be sufficient, thereby limiting radiation exposure if surgical intervention is unlikely. Additionally, recognizing the clinical signs and symptoms associated with acute infection of the temporal bone can help guide initial medical management in the emergency setting for situations when surgical intervention may be delayed or unnecessary. The literature suggests that uncomplicated mastoiditis, meaning no neurologic deficits or sepsis, should be managed with intravenous antibiotics, and CT temporal bone should only be obtained if deterioration or lack of clinical improvement is observed.^15^,^16^ Of note, no patients in our cohort had sepsis, which would have allowed us to further assess the utility of CT temporal bone specifically among patients with this acute concern. Operative intervention should be considered as a reasonable next step following medical management in such cases of disease progression or lack of improvement.^21^

In the setting of trauma evaluation, children are often exposed to significant radiation due to extensive radiologic workup. If initial findings on head CT suggest temporal bone fracture, a temporal bone CT may, in practice, be obtained to better visualize the fracture. Studies consistently indicate that facial nerve decompression is recommended in temporal bone fracture if the patient has complete facial nerve paralysis and a loss of greater than 90% function on ENoG.^10^,^22^ In such scenarios, temporal bone CT is useful in delineating the anatomical course of the facial nerve and locating the exact site of injury, guiding the decision on surgical approach (ie, trans-mastoid vs middle cranial fossa). Yet only 6% (N = 2) of trauma patients who underwent temporal bone CT in our study ultimately required facial nerve decompression. Even patients with incomplete facial nerve paralysis on initial presentation generally do well with expectant management.^10^

Surgical decompression for incomplete facial paralysis has a Grade D aggregate evidence level in the Otolaryngology – Head and Neck Surgery Clinical Practice Guidelines due to the lack of definitive benefits of surgery in this setting.^22^ Otherwise, temporal bone fractures are only monitored for hearing outcomes, which includes a delayed audiogram performed 3–6 weeks after injury to allow enough time for resolution of hemotympanum to accurately assess for ossicular discontinuity.^23^ Therefore, if there is no concern for complete facial nerve paralysis, a head CT alone obtained during routine trauma workup may be sufficient, since additional imaging is unlikely to change this management strategy and would increase radiation exposure. Following consultation with otolaryngology, consideration may be given for obtaining a temporal bone CT if there is concern for cerebrospinal fluid leak or otic capsule-violating fracture; however, even in these scenarios expectant management is often sufficient.^24^,^25^

It is worth noting that significant trauma is required to cause a temporal bone fracture, and many patients with such a finding have other more serious injuries taking precedence. Our algorithm (Figure 2) presupposes that no urgent/emergent neurosurgical pathology in or around the temporal bone is present, such as epidural hematoma or carotid canal injury, which may require additional imaging or other expedient management.

Previous studies have demonstrated that the radiologic finding of mastoid air cell opacification is non-specific, rarely clinically significant, and found incidentally in the pediatric population at rates of 14% and 21% with CT and magnetic resonance imaging (MRI), respectively.^26^,^27^ Additionally, Polat et al reported that only 17% of patients with mastoid opacification on MRI were found to have clinical infectious otologic disease.^28^ Therefore, despite the benefit of no radiation exposure with MRI, it is not necessarily superior to CT in ruling out this incidental finding. Furthermore, additional evaluation based on this finding can result in unnecessary treatment and expenditure of healthcare resources.^29^,^30^

Our study demonstrates that there were 68 total otolaryngology referrals for CT temporal bone findings among the patient cohort. We found that 94% of trauma patients and 23% of infectious patients for whom otolaryngology was consulted ultimately did not require operative intervention. Therefore, CT head findings should be closely correlated with clinical examination to reduce unnecessary temporal bone CT, and further imaging should be based on a coordinated approach between services when specialty consultation is requested. This descriptive study has identified trends for certain inefficiency and redundancy at our institution. With this data, we have developed algorithms to streamline the decision process in ordering temporal bone CT in the acute setting to foster shared decision-making between clinicians and specialties to ultimately reduce radiation exposure among the pediatric population and improve quality of patient care.

## LIMITATIONS

There are limitations to the findings of our study. First, this was a retrospective review performed at a single institution with a small sample size, and thus some of our conclusions may not be generalizable to other populations as practice patterns may vary by institution. Additionally, the chart abstractors were not blinded to the study hypothesis. While the study demonstrates an opportunity to reduce the decision to perform a CT, our study did not evaluate whether that decision was made by the ED, trauma surgery, or the consulting service. However, we hope that by highlighting this opportunity it will foster more collaboration between services in providing multidisciplinary care.

## CONCLUSION

Our study demonstrates that temporal bone CT can be beneficial in guiding diagnosis and management of acute infectious pathology in the pediatric emergency setting. Clinical examination findings such as mental status changes, proptosis, and fluctuance should guide decision-making surrounding the utility of temporal bone CT for mastoiditis. There may be circumstances in which imaging could be avoided to reduce radiation exposure. In traumatic pathology of the temporal bone, CT did not lead to operative intervention in patients without a clinically apparent facial nerve paralysis. We propose approaches to addressing CT imaging among pediatric patients with acute infectious concerns as well as those with temporal bone fractures, which emphasize using clinical findings likely to lead to operative intervention. By identifying scenarios in which imaging may be unnecessary, we hope to continue public health efforts to reduce ionizing radiation among the pediatric population.

## Figures and Tables

**Table 1:** Presenting signs, symptoms, radiographic findings, otolaryngology consults, and final diagnoses in trauma patients (N = 39) and patients with acute infectious concerns (N = 53).

	Trauma, N (%)	Infection, N (%)
Presenting symptoms		
Otalgia	6 (15)	42 (79)
Otorrhea	7 (18)	14 (26)
Hearing loss	7 (18)	6 (11)
Post-auricular pain / swelling	0 (0)	10 (19)
Presenting signs		
Mental status change	12 (31)	5 (9)
Acute otitis media	0 (0)	16 (30)
Mastoid tenderness	4 (10)	29 (55)
Hemotympanum	16 (41)	0 (0)
Auricular proptosis	0 (0)	17 (32)
Otorrhea	1 (3)	15 (28)
Bloody otorrhea	10 (26)	0 (0)
Abnormal tuning fork exam	9 (23)	2 (4)
Post-auricular erythema	0 (0)	10 (19)
Post-auricular fluctuance	0 (0)	6 (11)
Facial nerve paralysis	2 (5)	2 (4)
Radiographic findings		
No acute abnormality	2 (5)	10 (19)
Otic capsule-sparing fracture	29 (74)	0 (0)
Otic capsule-involving fracture	2 (5)	0 (0)
Mastoid effusion	16 (41)	19 (36)
Simple mastoiditis	0 (0)	11 (21)
Complicated mastoiditis	0 (0)	10 (19)
Otitis media	0 (0)	6 (11)
Otitis externa	0 (0)	8 (15)
Known cholesteatoma	0 (0)	5 (9)
Suspected cholesteatoma	0 (0)	1 (2)
Cavernous sinus thrombosis	0 (0)	1 (2)
Prior Head CT	29 (74)	5 (9)
Consults		
ENT Consult	33 (85)	35 (66)
Final diagnosis		
No acute ear pathology	2 (5)	9 (17)
Simple mastoiditis	0 (0)	6 (11)
Complicated mastoiditis	0 (0)	15 (28)
Simple otitis media	0 (0)	11 (21)
Complicated otitis media	0 (0)	2 (4)
Otitis externa	0 (0)	5 (9)
Eustachian tube dysfunction	0 (0)	1 (2)
Mastoid effusion	5 (13)	3 (6)
Temporal bone fracture	31 (79)	0 (0)
Cerumen impaction	1 (3)	1 (2)

*CT*, computed tomography.

*ENT*, ear, nose, and throat.

**Table 2:** Presenting signs, symptoms, and radiographic findings in patients with concern for mastoiditis, comparing the non-operative (N = 26) to the operative group (N = 27).

	Non-operative, N (%)	Operative, N (%)	*P*-value
Presenting symptoms			
Otalgia	22 (85)	20 (74)	0.74
Otorrhea	6 (23)	8 (30)	0.54
Hearing loss	4 (15)	2 (7)	0.67
Post-auricular pain	4 (15)	6 (22)	0.5
Mental status change	0 (0)	5 (19)	**0.02**
Presenting signs			
Tympanic membrane opacification	10 (38)	6 (22)	0.77
Mastoid tenderness	18 (69)	11 (41)	0.27
Auricular proptosis	4 (15)	13 (48)	**0.05**
Otorrhea	7 (27)	8 (30)	0.77
Post-auricular erythema	6 (23)	4 (15)	0.73
Post-auricular fluctuance	0 (0)	6 (22)	**0.02**
Facial nerve paralysis	0 (0)	2 (7)	0.24
Abnormal tuning fork exam	0 (0)	2 (7)	0.24
Radiographic findings			
Mastoid effusion	12 (46)	7 (26)	0.25
Simple mastoiditis	4 (15)	7 (26)	0.32
Complicated mastoiditis	0 (0)	10 (37)	<0.01
Prior Head CT	2 (8)	3 (11)	0.70
Consults			
ENT Consult	8 (31)	27 (100)	

*CT*, computed tomography; *ENT,* ear, nose, and throat.
